# Improved Generalizability in Medical Computer Vision: Hyperbolic Deep Learning in Multi-Modality Neuroimaging

**DOI:** 10.3390/jimaging10120319

**Published:** 2024-12-12

**Authors:** Cyrus Ayubcha, Sulaiman Sajed, Chady Omara, Anna B. Veldman, Shashi B. Singh, Yashas Ullas Lokesha, Alex Liu, Mohammad Ali Aziz-Sultan, Timothy R. Smith, Andrew Beam

**Affiliations:** 1 Harvard Medical School, Boston, MA 02115, USA; cyrusayubcha@hms.harvard.edu; 2 Department of Epidemiology, Harvard T.H. Chan School of Public Health, Boston, MA 02115, USA; 3 Department of Neurosurgery, Cushing Neurosurgical Outcomes Center, Brigham and Women’s Hospital, Harvard Medical School, Boston, MA 02115, USA; smsajed@bu.edu (S.S.); c.omara@lumc.nl (C.O.); a.b.veldman@lumc.nl (A.B.V.); asultan@bwh.harvard.edu (M.A.A.-S.); trsmith@bwh.harvard.edu (T.R.S.); 4 Department of Medical Science, Boston University, Boston, MA 02118, USA; 5 Department of Neurosurgery, Leiden University Medical Center, 2333 ZA Leiden, The Netherlands; 6 Stanford University School of Medicine, Stanford, CA 94305, USA; 7 Department of Radiology, Sri Devaraj Urs Medical College, Kolar 563101, India; dryashasullas@sduaher.ac.in; 8 Department of Computer and Information Sciences, Temple University, Philadelphia, PA 19122, USA

**Keywords:** hyperbolic neural networks, Euclidean, convolutional neural networks, Lorentz, neuroimaging, medical imaging, generalizability, adversarial robustness, hierarchical data structures

## Abstract

Deep learning has shown significant value in automating radiological diagnostics but can be limited by a lack of generalizability to external datasets. Leveraging the geometric principles of non-Euclidean space, certain geometric deep learning approaches may offer an alternative means of improving model generalizability. This study investigates the potential advantages of hyperbolic convolutional neural networks (HCNNs) over traditional convolutional neural networks (CNNs) in neuroimaging tasks. We conducted a comparative analysis of HCNNs and CNNs across various medical imaging modalities and diseases, with a focus on a compiled multi-modality neuroimaging dataset. The models were assessed for their performance parity, robustness to adversarial attacks, semantic organization of embedding spaces, and generalizability. Zero-shot evaluations were also performed with ischemic stroke non-contrast CT images. HCNNs matched CNNs’ performance in less complex settings and demonstrated superior semantic organization and robustness to adversarial attacks. While HCNNs equaled CNNs in out-of-sample datasets identifying Alzheimer’s disease, in zero-shot evaluations, HCNNs outperformed CNNs and radiologists. HCNNs deliver enhanced robustness and organization in neuroimaging data. This likely underlies why, while HCNNs perform similarly to CNNs with respect to in-sample tasks, they confer improved generalizability. Nevertheless, HCNNs encounter efficiency and performance challenges with larger, complex datasets. These limitations underline the need for further optimization of HCNN architectures. HCNNs present promising improvements in generalizability and resilience for medical imaging applications, particularly in neuroimaging. Despite facing challenges with larger datasets, HCNNs enhance performance under adversarial conditions and offer better semantic organization, suggesting valuable potential in generalizable deep learning models in medical imaging and neuroimaging diagnostics.

## 1. Background and Significance

Advances in computational power have facilitated the expansion of predictive deep learning models in many fields [1,2,3]. While deep learning models are idealized as universal approximators, not all tasks are not equally appropriate for all neural network architectures; as a result, misalignment may result in subpar empirical task performance [4]. One notable instance of this involves data structures with an inherent hierarchical structure [5]. Tree-like or hierarchical data structures have been shown to be represented with superior fidelity with less distortion in hyperbolic space compared to Euclidian space [6]. Specifically, the hyperbolic manifold allows for exponential scaling from the radial axis, which mirrors the distance relationships found in hierarchical structures and, accordingly, prevents distortion and information loss [7].

There has been a notable effort to utilize the superior data representation observed in hyperbolic spaces with deep learning algorithms. Over the last few years, many have attempted to operationalize constructs of hyperbolic space in a computationally efficient manner with the goal of reconstructing the fundamental functionality of neural network operations consistent with hyperbolic space [5]. Thus, hyperbolic neural networks (HNNs) were developed as an alternative to standard Euclidean neural networks [8]. While most papers explore densely connected HNNs, there have been efforts to use alternative neural network architectures. For instance, Khrulkov et al. developed a hybrid HNN that was applied to image datasets by utilizing a traditional Euclidean convolution structure and a connected layer prior to mapping the resulting embeddings into hyperbolic space and conducting a multi-class logistic regression [9]. Khrulkov et al. showed a superior performance to analogous Euclidean models in certain datasets (Caltech-UCSD Birds, DukeMTMC-reID dataset), as well as in few-shot classification [9]. They also showed that HNNs provided the more intuitive organization of the classes in the embedding space, likely explaining their improved out-of-sample and out-of-distribution performance [9]. 

Given the issues with numerical stability during training, most of the literature authors have attempted to make HNNs more numerically stable [10,11,12]. Guo et al. proposed a clipping mechanism that would bound the embedding space to coerce numerical stabilization during training [12]. These clipped HNNs were found to outperform standard HNNs in various benchmarks, including CIFAR10, CIFAR100, and ImageNet, and demonstrated better adversarial robustness and out-of-distribution detection [12]. Unlike unclipped HNNs, clipped HNNs achieved performance on par with ENNs in data settings without natural tree structures. The use of the Lorentz model of hyperbolics spaces, which has different mathematical constructions for the computational operation of the neural network compared to the Poincaré ball model, has also been proven to reduce numerical instability [11,13]. Finally, there have also been efforts to translate common Euclidian convolutional neural network operations into hyperbolic space, where the fully hyperbolic convolutional neural networks (HCNNs) could be contained in hyperbolic space in an end-to-end fashion [14]. This prevents the need for mapping between Euclidean and hyperbolic spaces, to limit the numerical instability.

Nevertheless, the literature remains uncertain in terms of fully exploring the value of HNNs. The original seminal paper in hyperbolic imaging embeddings suggests that most imaging datasets have some degree of implicit hierarchical structure [9]. Other studies found poor performance in settings without any natural hierarchical structures compared to Euclidean counterparts, until Guo et al. suggested that clipped HNNs could achieve similar performance in certain settings [12]. In summary, there is a clear need for further robust evaluation of HCNNs in various domains to weigh up the possible benefits and limitations of these evolving models.

One field of application in which the successful application of computer vision algorithms has been keenly appreciated is medical imaging [15]. To our knowledge, only one study has been performed utilizing HNNs for the classification of medical imaging data. Utilizing Khrulkov et al.’s hybrid paradigm, Yu et al. introduced a hyperbolic prototype network capable of jointly learning image embeddings and class prototypes in a shared hyperbolic space, guided by an error construction mechanism derived from a prior known class hierarchy [16]. Their approach preserved the semantic class relationships of dermatoscope images in the hyperbolic embedding space and found superior performance in classification compared to analogous Euclidean approaches, though with lower space curvature hyperparameters [16]. Other related works in generalizability for medical imaging include Bayesian approaches, which have leveraged hierarchical segmentation tasks with informed priors [17], as well as methods that have leveraged exploitability techniques such as Class Activation Mapping to optimize model generalizability [17,18]. 

Given the success of present-day convolutional neural networks in imaging recognition tasks, there has been increased interest in developing models that offer both a wide range of capabilities across a variety of tasks or classes and durability in a variety of clinical settings where models may encounter rare patient morphologies or imperfect images [19,20,21,22]. In the Materials and Methods section, we introduce the data sources, model architecture, and proposed experimental design. In the Results section, we report our experimental results, and in the Discussion section, we highlight the novel results of our work and how they relate to the prior literature in geometric deep learning and medical computer vision. Our contributions are as follows:

- We explore the value of HCNNs by evaluating the classification performance of clipped HCNNs relative to their Euclidean counterparts;
- Agnostic to any prior data hierarchy, we conduct an evaluation of in-sample performance across three medical imaging datasets of varying complexity to evaluate model performance with increasing task complexity;
- We evaluate the models by their organization of embedding spaces;
- We explore generalizability in the form of durability against adversarial attacks and out-of-sample and zero-shot performance.

## 2. Materials and Methods

### 2.1. Data Sources

We developed a core neuroimaging dataset to evaluate the HCNN and CNN models: the Multi-Modality Neuroimaging (MMN) Dataset, composed of 72,634 images of 42 total classes. The images were acquired from previously open-source databases, which included various neurological diseases, including ischemic stroke [23], hemorrhagic stroke [24], metastasis [25], tumor [26], schizophrenia (COBRE, MCICShare) [27], and Alzheimer’s disease (AD) [28], across computed tomography (CT) and magnetic resonance imaging (MRI) modalities. Images that were not from a peer-reviewed source were independently confirmed to be correctly classified by a qualified radiologist. 

Certain classes in the neuroimaging dataset were manually composed either due to overlapping classes or, occasionally, to better capture disease signals. Specifically, the “normal” categories were composed of multiple appropriate non-diseased classes in the datasets above. For instance, patients in the schizophrenia databases defined as “normal” patients were moved into the respective normal classes once we excluded the possibility of them joining another diseased class in the database. Schizophrenia-positive scans were restricted to four of the median axial scans for each patients to better capture morphological abnormalities commonly conferred by patients with schizophrenia [29].

We also constructed two additional datasets of other medical imaging types to conduct further analyses in larger and smaller class settings: the Miniature Multi-Disease Dataset (MMD) and the Multi-Disease Dataset (MD). The MD is composed of 89,496 images of 78 total classes. These images were acquired from previously published open-source databases, which include Chest X-Rays [30], Fundoscopy [31], Gastrointestinal Scopes [32], Musculoskeletal X-Rays [33], Neuroimaging, and Dermatoscopy [34,35,36]. Finally, the MMD was restricted to a smaller subset of the MMD with a total of 19,880 unique images across 16 total classes. We included the largest publicly available structural imaging datasets that were available for the respective diseases that conferred axial images and provided a “normal” class of data. The identity and balance of the classes and their respective image counts can be observed in Table 1. 

Out-of-sample data were acquired from the T-1 MRI sequences of the OASIS-1 cross-sectional cohort imaging study [37]. We derived one mid-brain axial slice from each patient in the study and defined an AD-positive case as an individual with a Mini-Mental State Examination (MMSE) score of below 25 and negative otherwise. In total, this dataset entailed a total of 436 subjects, where 40 subjects were AD-positive and 396 were AD-negative. The zero-shot dataset was acquired from the Mass General Brigham (MGB) Research Patient Data Registry (RPDR) with MGB Institutional Review Board approval. We retrospectively selected individuals from January 2004 to December 2022 who received a non-contrast computed tomography (NCCT) brain scan upon presenting to the emergency department. We further restricted ourselves to those diagnosed with acute ischemic strokes via magnetic resonance imaging (MRI) brain scans within 7 days of their original presentation to the emergency department. We included the full axial scans of the 151 patients for a total of 23,371 images. We also documented the radiologist reports and their ability to correctly able to diagnose the ischemic stroke on NCCT. We labelled the axial images as positive if they included regions implicated by the positive MRI reads for that patient where all other images were deemed negative for that patient. 

### 2.2. Processing and Models

All training data underwent uniform pre-processing transformations prior to their use in the model, including normalization, rotation, and horizontal flip. Furthermore, to homogenize the diverse presentation of brain imaging data, we conducted skull-stripping when appropriate. For all the models in this study, we utilized a Res-Net-18 (RN18) backbone, where we defined the baseline Euclidean CNN as an RN18 model. The HCNN models were constructed as hybrid models with an identical convolutional structure to the CNN. However, the HCNN model acquired the embeddings from the convolutional encoder and translated them into the Lorentz space with an exponent mapping procedure, where the remaining operations of the RN18-based decoder resided. For the Lorentz models, a Riemannian SGD optimizer, a learnable Lorentz decoder curvature parameter (k), and a clipped feature constraint (1.0) were employed. More details on our hybrid HCNN construct, including trainable curvature, feature-clipping [12], and Euclidean reparameterizations [13], which were documented by the Bdeir et al. [38] code base, were utilized in our study. Please refer to the Supplementary Material for our code implementation. 

### 2.3. Evaluation

To examine the performance of each model in the respective dataset, we reproduced the cross-entropy loss, Top-1 accuracy, and Top-5 accuracy. We also derived the embedding space for each model, leveraging the n-dimension space, where n represented the number of classes in the respective model prior to the SoftMax and output layers. Utilizing the average position within this embedding space for each class, we constructed a low-dimensional representation using T-SNE algorithms with the Euclidean and Lorentz models, respectively. We also utilized hierarchical clustering procedures with the average class embedding positions for each model, allowing us to derive a dendrogram of the inter-class relationships learned by the respective models. 

To examine the interpretability of the embedding space, we compared the geodesic distance matrices of the average class embeddings from the respective Euclidean and Euclidean–Lorentz models to a ground truth hierarchical distance matrix. We derived the ground truth distance matrix based on the sets of categorical variables as descriptors of the imaging category and disease type (refer to Table S1). We first normalized all distance matrices and computed the absolute pairwise difference between the model distance matrix and the ground truth distance matrix. We also derived Spearman’s rank correlation coefficient between the respective model and ground truth distances matrices based on the ranked distance of the pairwise classes. 

To evaluate the out-of-sample accuracy, we utilized a single median axial slice in each OASIS-1 subject and considered a positive diagnosis as one that ascribed an AD-related class to the scan and a negative diagnosis as a normal T1 MRI predicted class. Next, we evaluated the zero-shot accuracy by defining a true-positive diagnosis read as having an axial NCCT image predicted as an ischemic or hemorrhagic stroke class by the model where the ground truth was positive. At a patient level, we defined accuracy as a patient having at least one true positive. We defined a true negative as a normal CT class. Then, we identified the overall image-based accuracy as the number of true positives and true negatives over the overall number of images. Finally, we conducted a series of Projected Gradient Descent (PGD) adversarial attacks to assess the comparative durability of each model against distortions in data. For each model, we conducted three separate attacks with increasing epsilon values (0.03, 0.06, 0.12), and we present the resultant top accuracy from the converged models. Note that all 95% confidence intervals are derived from a respective bootstrapping procedure (*n* = 1000).

## 3. Results

**Figure 1:**
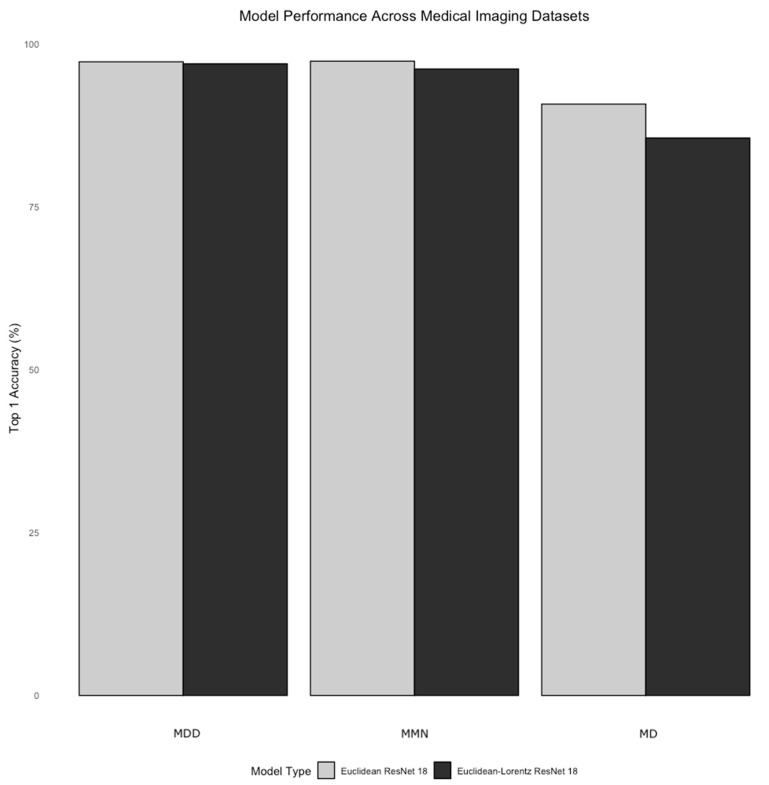
Relative model performance across datasets. The bar plot above shows the Top-1 accuracy metrics with 95% confidence intervals for the Euclidean ResNet 18 and the Euclidean–Lorentz ResNet 18 across the three datasets, increasing in size from left to right (i.e., Miniature Multi-Disease (MDD) Dataset, Multi-Modality Neuroimaging (MMN) Dataset, and Multi-Disease (MD) Dataset).

**Figure 2:**
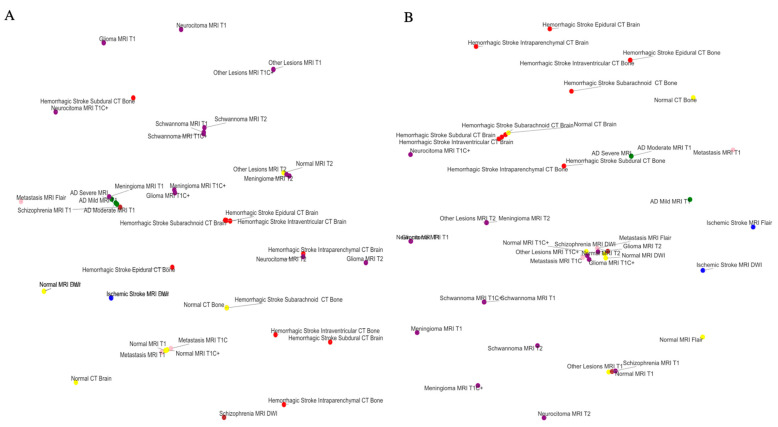
Euclidean and hyperbolic model T-SNE in the Neuroimaging Dataset. This Figure shows the low-dimensional representation T-SNE of the average class embedding space from the Euclidean ResNet 18 (**A**) and the Euclidean–Lorentz ResNet 18 (**B**) for the Multi-Modality Neuroimaging (MMN) Dataset. The colors denote the broader category per class.

**Figure 3:**
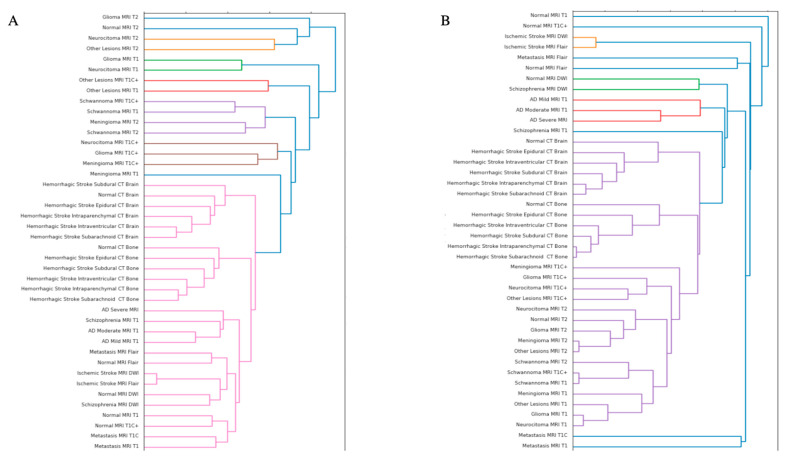
Euclidean and hyperbolic model dendrograms for the Neuroimaging Dataset. This Figure illustrates the hierarchical clustering dendrogram of the average class embedding space of the Euclidean ResNet 18 (**A**) and the Euclidean–Lorentz ResNet 18 (**B**) for the Multi-Modality Neuroimaging (MMN) Dataset.

The performance of the Euclidean model was generally matched by the Euclidean–Lorentz model in the MMN and the MMDs with minimal differences in Top-1 accuracy and identical Top-5 accuracy (Table 2). More broadly, however, the Euclidean model generally began to outperform the Euclidean–Lorentz model as the number of images and the class size of the dataset increased; this is most prominent in the MD dataset (Figure 1). Interpreting the low-dimensional T-SNE representation of the average embedding class, as well as the respective dendrogram, the Euclidean–Lorentz model appears to have a more reasonable distribution of embedding space based on prior understanding of the inter-relationships between the imaging classes in the MMN (Figure 2 and Figure 3). Clustering results for other imaging datasets can be found in the Figures S1–S3. 

When compared to the known ground truth distance matrix, the distinction between the two models becomes more apparent. The mean absolute difference between the respective Euclidean and Euclidean–Lorentz models compared to the ground truth distance matrix for the MMN dataset was highest in the Euclidean model (0.290 ± 0.005). In contrast, the mean absolute difference in the Euclidean–Lorentz model was significantly lower (0.158 ± 0.003), with a two-sample *t*-test *p*-value of <0.0001. Spearman’s rank correlation findings show that the Euclidean model exhibited a weak correlation with the ground truth ranking (correlation coefficient = 0.021, *p* = 0.3783), while the Euclidean–Lorentz model showed a stronger correlation (correlation coefficient = 0.328, *p* < 0.0001). These results indicate that the Euclidean–Lorentz model not only has a lower mean absolute difference compared to the ground truth but also demonstrates a stronger correlation to the ground truth class ranks.

**Figure 4:**
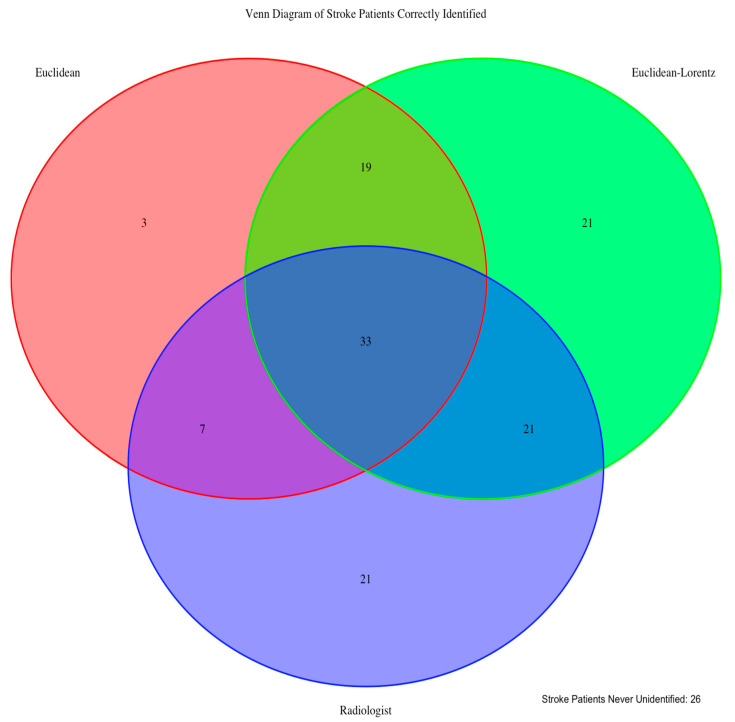
Zero-shot identification of stroke patients. The diagram above shows how many of the zero-shot stroke patients were identified across the Euclidean and Euclidean–Lorentz models, as well as by human radiologists with emergent non-contrast brain CT imaging. We also note that 26 patients were not identified using any of the three approaches.

Compared to the radiologist’s performance, which identified 82 of the 151 patients (0.53), the Euclidean model performed worse, identifying only 62 stroke patients (0.41), while the Euclidean–Lorentz model outperformed by identifying 94 (0.62). Across all images, the Euclidean–Lorentz model achieved a higher overall accuracy (0.50) than the Euclidean model (0.45) (Figure 4).

In the out-of-sample dataset, both models were able to correctly identify the modality of the axial images with a 100% identification rate. The Euclidean–Lorentz model and the Euclidean model achieved a Top-1 accuracy of 0.54 (95% CI: 0.44, 0.64) and 0.55 (95% CI: 0.45, 0.65), respectively, suggesting a statistically indistinguishable performance in this out-of-sample dataset. Within the NCCT ischemic stroke dataset, the negative cases were technically a class already observed by the models, so we used this as an additional out-of-sample experiment, where the models were tasked with correctly identifying negative NCCT slices as normal CT images. The Euclidean model achieved an accuracy for the negative axial NCCT images of 0.81 (95% CI: 0.81–0.82), which was statistically comparable to the Euclidean–Lorentz model, which reached an accuracy of 0.82 (95% CI: 0.82–0.83).

Interestingly, the PGD adversarial attack analysis suggests that the Euclidean–Lorentz model often outperforms its Euclidean counterpart in the larger MMN and MD datasets with respect to the Top-1 and Top-5 accuracy metrics (Table 3). The performance becomes more similar across the two models in the smaller MMD.

## 4. Discussion

Limitations in model generalizability are a significant barrier to the large-scale clinical implementation of deep learning methods in medical imaging settings. Our empirical analysis study elucidates several important insights into the comparative value of clipped Euclidean–Lorentz HCNNs and Euclidean CNNs in neuroimaging tasks, as well as other medical imaging settings, especially with respect to generalizability. The results suggest parity in performance between the two neural network approaches in smaller, less complex datasets. We further note distinct semantic organization within the respective embedding spaces, with the HCNN aligning better with ground truth relations between the neuroimaging classes. In assessing generalizability, the HCNN achieved a similar out-of-sample performance in identifying AD and normal NCCT images but a greatly improved zero-shot performance in identifying ischemic stroke in NCCT images.

The cross-entropy loss and Top-1 accuracy metrics followed a similar trend across the three medical imaging datasets. Notably, these metrics were identical or similar in datasets where the CNN achieved a higher performance (>95% accuracy). However, as the complexity and size of the datasets grew, there was a precipitous drop in HCNN performance compared to the CNN. Interestingly, despite the difference in loss in the MMN dataset, the Top-1 accuracy between the two models was more similar, unlike in the MD dataset. In the settings of both performance parity and disparity, the Top-5 accuracy metric across the two models was nearly identical in all three datasets, perhaps due to the improved generalizability of HCNNs, which we will explore further.

The achievement of parity replicates the findings from Guo et al., which demonstrate that clipped HCNNs achieve a similar performance in data settings without strong hierarchy [12]. Nevertheless, we illustrate that the performance of the HCNN suffers compared to the CNN when applied to larger datasets with seemingly more difficult tasks. Given the similarity in model size across the three datasets, our findings may suggest that HCNNs, as currently constructed, are less efficient with their trainable parameters, contrary to the prior literature [8]. 

One of the known features of HCNNs is the improved preservation of hierarchical data structures, as reflected by the organized embedding space [5,9]. Low-dimensional T-SNE representations of the embedding space suggest a stratification of classes in the MMN dataset, often by modality first and then disease type, in both models. Similarities in class grouping may be starker in the Euclidean–Lorentz model, as observed in the hierarchical clustering dendrogram from the embedding space. 

Nevertheless, the noted limitations of low-dimensional representations may offer a distorted view of the true geodesic distances between the average class embeddings [39]. To explore whether the two models developed meaningfully distinct organizations of embedding space in a more robust fashion, we derived a respective geodesic distance matrix for the average class embedding in both MMN models. We then compared the pairwise distance matrix from each model against a constructed ground truth difference matrix between the classes. Using the pairwise distance differences, as well as Spearman’s rank correlation coefficient, we showed that the HCNN, and not the CNN, better aligned with our known semantic understanding of the class relationships.

As we observed superior learning and conservation of known class structures in neuroimaging data, we further explored the tangible value of this distinguishing feature. One of the most important aspects of any diagnostic medical imaging algorithm is its ability to function with out-of-sample and out-of-distribution imaging data [40]. We specifically found that the MMN HCNN performed similarly to the CNN in the OASIS I and stroke-negative NCCT datasets, despite a poorer HCNN performance in terms of Top-1 accuracy and loss in the MMN dataset.

We also found that in terms of zero-shot performance, the HCNN unequivocally outperformed not only the CNN but also the trained radiologists by a significant margin. Finally, the HCNN showed consistently increased durability to adversarial attacks, which may be relevant in terms on confrontation with imaging artifacts, image quality disparities between scanners, or image corruption that may or may not be perceptible [41,42]. As suggested by prior studies in non-medical imaging settings [9,11,12], we showed that the neuroimaging HCNN improved generalizability with respect to out-of-sample, zero-shot, and adversarial attack performance.

Our study should be interpreted as having certain limitations. While recent studies have attempted to move convolutional functions into hyperbolic space [14,38], we observed significant numerical instability with these methods. While we did not observe numerical instability in the hybrid models used in this study, the computational efficiency of HCNNs was observed to be dramatically lower, with convergence requiring three to four times more epochs with similar hyperparameters. This was even more pronounced in larger datasets, limiting our ability to use larger datasets. We tested task complexity and size concurrently across the three datasets, but future work should further explore scalability and complexity. Additionally, we are restricted to speculating on the performance of clipped hybrid HCNNs in the context of our included medical imaging classes and can only discuss generalizability in terms of the out-of-sample and zero-shot datasets used.

These present limitations may slow down the larger-scale applications with larger training sets that are required in models applied to clinical settings. As such, further research and development is needed to improve the efficiency and scope of these hyperbolic models. Alternatively, other methods for achieving better generalizability, including federated models and the complication of diverse datasets, continue to represent a valuable practical approach [43,44].

## 5. Conclusions

In this study, we showed that agnostic HCNN models demonstrated a superior ability to learn and retain a native hierarchical structure in a neuroimaging dataset compared to Euclidean CNNs. Importantly, the HCNN achieved a disproportionately superior performance in adversarial attack experiments and zero-shot settings, outperforming both radiologists and CNNs. The neuroimaging HCNN also achieved parity in in-sample performance with out-of-sample data but showed a depreciating performance in more complex medical imaging task settings with larger datasets. These findings suggest that improvements in the efficiency and scalability of HCNNs are needed to achieve parity with CNNs. However, HCNNs provided notable value in their generalizability in medical imaging settings with multi-modal and multi-disease neuroimaging data.

## Figures and Tables

**Table 1:** Dataset characteristics.

Miniature Multi-Disease Dataset: Classes	Images	Multi-Modality Neuroimaging Dataset: Classes	Images	Multi-Disease Dataset: Classes	Images
Derm: Actinic Keratosis	867	AD Moderate MRI T1	896	Derm: Actinic Keratosis	867
Derm: Basal Cell Carcinoma	3323	AD Severe MRI	64	Pulm: Bacterial Pneumonia	2780
Derm: Benign Keratosis	2624	AD Mild MRI T1	2240	Derm: Basal Cell Carcinoma	3323
Derm: Dermatofibroma	239	Hemorrhagic Stroke Epidural CT Bone	167	Derm: Benign Keratosis	2624
Derm: Melanoma	4522	Hemorrhagic Stroke Intraparenchymal CT Bone	52	Derm: Dermatofibroma	239
Derm: Melanocytic Nevi	12,875	Hemorrhagic Stroke Intraventricular CT Bone	13	Neuro: Epidural Hemorrhagic Stroke CT Bone	167
Derm: Squamous Cell Carcinoma	628	Hemorrhagic Stroke Subarachnoid CT Bone	9	Neuro: Intraparenchymal Hemorrhagic Stroke CT Bone	52
Derm: Vascular Lesion	253	Hemorrhagic Stroke Subdural CT Bone	52	Neuro: Subdural Hemorrhagic Stroke CT Bone	52
Gastro: Dyed Lifted Polyps	1000	Hemorrhagic Stroke Epidural CT Brain	167	Neuro: Epidural Hemorrhagic Stroke CT Brain	167
Gastro: Dyed Resection Margins	1000	Hemorrhagic Stroke Intraparenchymal CT Brain	52	Neuro: Intraparenchymal Hemorrhagic Stroke CT Brain	52
Gasto: Esophagitis	1000	Hemorrhagic Stroke Intraventricular CT Brain	13	Neuro: Subdural Hemorrhagic Stroke CT Brain	52
Optho: Normal Fundus	2873	Hemorrhagic Stroke Subarachnoid CT Brain	9	Neuro: Ischemic Stroke DWI	1012
Gastro: Normal Cecum	1000	Hemorrhagic Stroke Subdural CT Brain	52	Neuro: Ischemic Stroke Flair	1002
Gastro: Normal Pylorus	1000	Ischemic Stroke MRI DWI	1012	Derm: Melanoma	4522
Gasto: Normal Z-Line	1000	Ischemic Stroke MRI Flair	1002	Neuro: Metastasis Flair	4248
Gastro: Polyps	1000	Metastasis MRI Flair	4248	Neuro: Metastasis T1	4248
		Metastasis MRI T1C	4248	Neuro: Metastasis T1C+	4248
		Metastasis MRI T1	4248	Pulm: Normal CXR	1583
		Normal CT Bone	1495	Derm: Melanocytic Nevi	12,875
		Normal CT Brain	1494	Neuro: Normal CT Bone	1495
		Normal MRI DWI	1406	Neuro: Normal CT Brain	1494
		Normal MRI Flair	14,949	Neuro: Normal DWI	1406
		Normal MRI T1	17,925	Neuro: Normal_Flair	4949
		Normal MRI T1C+	13,941	Neuro: Normal T1	7925
		Normal MRI T2	18	Neuro: Normal T1C+	3941
		Schizophrenia MRI DWI	471	Neuro: Normal T2	18
		Schizophrenia MRI T1	1314	Derm: Squamous Cell Carcinoma	628
		Glioma MRI T1C+	152	Neuro: Schizophrenia DWI	471
		Meningioma MRI T1C+	233	Neuro: Schizophrenia T1	1314
		Neurocitoma MRI T1C+	76	Neuro: Glioma T1	152
		Other Lesions MRI T1C+	9	Neuro: Meningioma T1	233
		Schwannoma MRI T1C+	36	Neuro: Neurocitoma T1	76
		Glioma MRI T1	65	Neuro: Schwannoma T1	36
		Meningioma MRI T1	141	Neuro: Glioma T1C+	65
		Neurocitoma MRI T1	39	Neuro: Meningioma T1C+	141
		Other Lesions MRI T1	27	Neuro: Neurocitoma T1C+	39
		Schwannoma MRI T1	31	Neuro: Schwannoma T1C+	31
		Glioma MRI T2	9	Neuro: Glioma T2	67
		Meningioma MRI T2	67	Neuro: Meningioma T2	145
		Neurocitoma MRI T2	145	Neuro: Schwannoma T2	33
		Other Lesions MRI T2	14	Derm: Vascular Lesion	253
		Schwannoma MRI T2	33	Pulm: Viral Pneumonia	1493
				Optho: Branch Retinal Vein Occlusion	19
				Optho: Cataract	287
				Optho: Diabetic Retinopathy	53
				Optho: Drusen	148
				Optho: Dry Age-Related Macular Degeneration	202
				Gastro: Dyed Lifted Polyps	1000
				Gastro: Dyed Resection Margins	1000
				Optho: Epiretinal Membrane	140
				Gasto: Esophagitis	1000
				Optho: Glaucoma	213
				MSK: Hand Normal	877
				MSK: Hand Fractured	379
				MSK: Hand Shoulder Normal	180
				MSK: Hand Shoulder Fractured	53
				MSK: Hip Normal	169
				MSK: Hip Fractured	13
				Optho: Hypertensive Retinopathy	123
				Optho: Macular Epiretinal Membrane	140
				Optho: Maculopathy	23
				Optho: Mild Nonproliferative Retinopathy	464
				Optho: Moderate Non-Proliferative Retinopathy	798
				Optho: Myelinated Nerve Fibers	68
				Optho: Normal Fundus	2873
				Gastro: Normal Cecum	1000
				Gastro: Normal Pylorus	1000
				Gasto: Normal Z-Line	1000
				Optho: Pathological Myopia	231
				Gastro: Polyps	1000
				Optho: Refractive Media Opacity	54
				Optho: Severe Nonproliferative Retinopathy	144
				Gastro: Ulcerative Colitis	1000
				Optho: Vitreous Degeneration	58
				Optho: Wet Age-Related Macular Degeneration	41

**Table 2:** Model characteristics and performance. The bold denotes the better performing model.

Dataset	Class Size	Sample Size	Model Type	Trainable Parameters	Cross Entropy Loss	Top 1 Accuracy	Top 1 Accuracy: 95% Confidence Intervals	Top 5 Accuracy
MMN	42	72,634	Euclidean ResNet 18	11,189,226	**0.05**	**97.4**	**97.3–97.5**	**99.9**
MMN	42	72,634	Euclidean–Lorentz ResNet 18	11,189,227	0.12	96.2	96.1–96.4	**99.9**
MD	75	89,496	Euclidean ResNet 18	11,207,307	**0.26**	**90.8**	**90.6–91.0**	**99.6**
MD	75	89,496	Euclidean–Lorentz ResNet 18	11,207,308	0.47	85.6	85.4–85.9	99.0
MMD	16	19,880	Euclidean ResNet 18	11,177,040	**0.11**	**97.3**	**97.1–97.4**	**99.9**
MMD	16	19,880	Euclidean–Lorentz ResNet 18	11,177,041	**0.11**	97.0	**96.8–97.1**	**99.9**

**Table 3:** Projected Gradient Descent adversarial attack. The bold denotes the better performing model.

Dataset	Model Type	Epsilon	Top 1 Accuracy	Top 5 Accuracy
MMN	Euclidean ResNet 18	0.003	6.50	53.12
MMN	Euclidean–Lorentz ResNet 18	0.003	**45.72**	**91.15**
MMN	Euclidean ResNet 18	0.006	0.08	6.55
MMN	Euclidean–Lorentz ResNet 18	0.006	**4.87**	**41.91**
MMN	Euclidean ResNet 18	0.012	0.00	2.81
MMN	Euclidean–Lorentz ResNet 18	0.012	**0.52**	**18.37**
MD	Euclidean ResNet 18	0.003	3.78	56.38
MD	Euclidean–Lorentz ResNet 18	0.003	**12.47**	**69.74**
MD	Euclidean ResNet 18	0.006	0.02	14.55
MD	Euclidean–Lorentz ResNet 18	0.006	**2.64**	**38.03**
MD	Euclidean ResNet 18	0.012	0.01	3.50
MD	Euclidean–Lorentz ResNet 18	0.012	**0.17**	**23.16**
MMD	Euclidean ResNet 18	0.003	13.48	**74.63**
MMD	Euclidean–Lorentz ResNet 18	0.003	**13.93**	48.04
MMD	Euclidean ResNet 18	0.006	1.29	**33.55**
MMD	Euclidean–Lorentz ResNet 18	0.006	**2.22**	28.92
MMD	Euclidean ResNet 18	0.012	0.01	16.05
MMD	Euclidean–Lorentz ResNet 18	0.012	**0.27**	**23.95**

## Data Availability

All publicly available data is cited in the methods section. MGB data is unable to be shared due to health data privacy regulations.

## Supplementary Materials

The following supporting information can be downloaded at: https://www.mdpi.com/article/10.3390/jimaging10120319/s1, Table S1: Neuroimaging Class Table Characteristics Tables; Figure S1: Average Class Embedding Space Distance Heat Map; Figure S2: Hierarchical Dendrograms in the Miniature Multi-Disease Dataset; Figure S3: Hierarchical Dendrograms in the Multi-Disease Dataset; Code Implementation.

## References

[1] Ren B., Liu M., Ding R., Liu H. (2024). A Survey on 3D Skeleton-Based Action Recognition Using Learning Method. Cyborg Bionic Syst..

[2] Qian K., Bao Z., Zhao Z., Koike T., Dong F., Schmitt M., Dong Q., Shen J., Jiang W., Jiang Y. (2024). Learning Representations from Heart Sound: A Comparative Study on Shallow and Deep Models. Cyborg Bionic Syst..

[3] Han Z., Tian H., Han X., Wu J., Zhang W., Li C., Qiu L., Duan X., Tian W. (2024). A Respiratory Motion Prediction Method Based on LSTM-AE with Attention Mechanism for Spine Surgery. Cyborg Bionic Syst..

[4] Lu Y., Lu J. (2020). A Universal Approximation Theorem of Deep Neural Networks for Expressing Probability Distributions. Adv. Neural Inf. Process. Syst..

[5] Ganea O., Becigneul G., Hofmann T. (2018). Hyperbolic Neural Networks. Adv. Neural Inf. Process. Syst..

[6] Sala F., Sa C.D., Gu A., Re C. Representation Tradeoffs for Hyperbolic Embeddings. Proceedings of the 35th International Conference on Machine Learning.

[7] Sarkar R., van Kreveld M., Speckmann B. (2012). Low Distortion Delaunay Embedding of Trees in Hyperbolic Plane. Graph Drawing.

[8] Peng W., Varanka T., Mostafa A., Shi H., Zhao G. (2022). Hyperbolic Deep Neural Networks: A Survey. IEEE Trans. Pattern Anal. Mach. Intell..

[9] Khrulkov V., Mirvakhabova L., Ustinova E., Oseledets I., Lempitsky V. Hyperbolic Image Embeddings. Proceedings of the 2020 IEEE/CVF Conference on Computer Vision and Pattern Recognition (CVPR).

[10] Shimizu R., Mukuta Y., Harada T. (2021). Hyperbolic Neural Networks++. arXiv.

[11] Chen W., Han X., Lin Y., Zhao H., Liu Z., Li P., Sun M., Zhou J. Fully Hyperbolic Neural Networks. Proceedings of the 60th Annual Meeting of the Association for Computational Linguistics (Volume 1: Long Papers).

[12] Guo Y., Wang X., Chen Y., Yu S.X. Clipped Hyperbolic Classifiers Are Super-Hyperbolic Classifiers. Proceedings of the 2022 IEEE/CVF Conference on Computer Vision and Pattern Recognition (CVPR).

[13] Mishne G., Wan Z., Wang Y., Yang S. The Numerical Stability of Hyperbolic Representation Learning. Proceedings of the 40th International Conference on Machine Learning.

[14] van Spengler M., Berkhout E., Mettes P. Poincaré ResNet. Proceedings of the 2023 IEEE/CVF International Conference on Computer Vision (ICCV).

[15] Rana M., Bhushan M. (2023). Machine learning and deep learning approach for medical image analysis: Diagnosis to detection. Multimed. Tools Appl..

[16] Yu Z., Nguyen T., Gal Y., Ju L., Chandra S.S., Zhang L., Bonnington P., Mar V., Wang Z., Ge Z. (2022). Skin Lesion Recognition with Class-Hierarchy Regularized Hyperbolic Embeddings. Medical Image Computing and Computer Assisted Intervention—MICCAI 2022.

[17] Gao S., Zhou H., Gao Y., Zhuang X. (2023). BayeSeg: Bayesian modeling for medical image segmentation with interpretable generalizability. Med. Image Anal..

[18] Chaddad A., Hu Y., Wu Y., Wen B., Kateb R. (2024). Generalizable and explainable deep learning for medical image computing: An overview. Curr. Opin. Biomed. Eng..

[19] Litjens G., Kooi T., Bejnordi B.E., Setio A.A.A., Ciompi F., Ghafoorian M., van der Laak J.A.W.M., van Ginneken B., Sánchez C.I. (2017). A survey on deep learning in medical image analysis. Med. Image Anal..

[20] Shi P., Qiu J., Abaxi S.M.D., Wei H., Lo F.P.-W., Yuan W. (2023). Generalist Vision Foundation Models for Medical Imaging: A Case Study of Segment Anything Model on Zero-Shot Medical Segmentation. Diagnostics.

[21] Li X., Zhang L., Wu Z., Liu Z., Zhao L., Yuan Y., Liu J., Li G., Zhu D., Yan P. (2023). Artificial General Intelligence for Medical Imaging. arXiv.

[22] Hirano H., Minagi A., Takemoto K. (2021). Universal adversarial attacks on deep neural networks for medical image classification. BMC Med. Imaging.

[23] Tasci B., Tasci I. (2022). Deep feature extraction based brain image classification model using preprocessed images: PDRNet. Biomed. Signal Process. Control.

[24] Hssayeni M. (2019). Computed Tomography Images for Intracranial Hemorrhage Detection and Segmentation. PhysioNet.

[25] Grøvik E., Yi D., Iv M., Tong E., Rubin D., Zaharchuk G. (2020). Deep learning enables automatic detection and segmentation of brain metastases on multisequence MRI. J. Magn. Reson. Imaging.

[26] Brain Tumor MRI Images 17 Classes. https://www.kaggle.com/datasets/fernando2rad/brain-tumor-mri-images-17-classes.

[27] Ambite J.L., Tallis M., Alpert K., Keator D.B., King M., Landis D., Konstantinidis G., Calhoun V.D., Potkin S.G., Turner J.A., Ashish N., Ambite J.-L. (2015). SchizConnect: Virtual Data Integration in Neuroimaging. Data Integration in the Life Sciences.

[28] Hernandez R.M., Sison D.K., Nolasco N.C., Melo J., Castillo R. Application of Machine Learning on MRI Scans for Alzheimer’s Disease Early Detection. Proceedings of the 8th International Conference on Sustainable Information Engineering and Technology.

[29] Shenton M.E., Dickey C.C., Frumin M., McCarley R.W. (2001). A review of MRI findings in schizophrenia. Schizophr. Res..

[30] Kermany D.S., Goldbaum M., Cai W., Valentim C.C.S., Liang H., Baxter S.L., McKeown A., Yang G., Wu X., Yan F. (2018). Identifying Medical Diagnoses and Treatable Diseases by Image-Based Deep Learning. Cell.

[31] Li N., Li T., Hu C., Wang K., Kang H. (2021). A Benchmark of Ocular Disease Intelligent Recognition: One Shot for Multi-disease Detection. arXiv.

[32] Pogorelov K., Randel K.R., Griwodz C., Eskeland S.L., de Lange T., Johansen D., Spampinato C., Dang-Nguyen D.-T., Lux M., Schmidt P.T. KVASIR: A Multi-Class Image Dataset for Computer Aided Gastrointestinal Disease Detection. Proceedings of the 8th ACM on Multimedia Systems Conference.

[33] Abedeen I., Rahman M.A., Prottyasha F.Z., Ahmed T., Chowdhury T.M., Shatabda S. (2023). FracAtlas: A Dataset for Fracture Classification, Localization and Segmentation of Musculoskeletal Radiographs. Sci. Data.

[34] Tschandl P., Rosendahl C., Kittler H. (2018). The HAM10000 dataset, a large collection of multi-source dermatoscopic images of common pigmented skin lesions. Sci. Data.

[35] Codella N.C.F., Gutman D., Celebi M.E., Helba B., Marchetti M.A., Dusza S.W., Kalloo A., Liopyris K., Mishra N., Kittler H. Skin Lesion Analysis Toward Melanoma Detection: A Challenge at the 2017 International Symposium on Biomedical Imaging (ISBI), Hosted by the International Skin Imaging Collaboration (ISIC). Proceedings of the 2018 IEEE 15th International Symposium on Biomedical Imaging (ISBI 2018).

[36] Combalia M., Codella N.C.F., Rotemberg V., Helba B., Vilaplana V., Reiter O., Carrera C., Barreiro A., Halpern A.C., Puig S. (2019). BCN20000: Dermoscopic Lesions in the Wild. arXiv.

[37] Marcus D.S., Wang T.H., Parker J., Csernansky J.G., Morris J.C., Buckner R.L. (2007). Open Access Series of Imaging Studies (OASIS): Cross-sectional MRI Data in Young, Middle Aged, Nondemented, and Demented Older Adults. J. Cogn. Neurosci..

[38] Bdeir A., Schwethelm K., Landwehr N. (2024). Fully Hyperbolic Convolutional Neural Networks for Computer Vision. arXiv.

[39] Sorzano C.O.S., Vargas J., Montano A.P. (2014). A survey of dimensionality reduction techniques. arXiv.

[40] Finlayson S.G., Bowers J.D., Ito J., Zittrain J.L., Beam A.L., Kohane I.S. (2019). Adversarial attacks on medical machine learning. Science.

[41] Apostolidis K.D., Papakostas G.A. (2021). A Survey on Adversarial Deep Learning Robustness in Medical Image Analysis. Electronics.

[42] Shi X., Peng Y., Chen Q., Keenan T., Thavikulwat A.T., Lee S., Tang Y., Chew E.Y., Summers R.M., Lu Z. (2022). Robust convolutional neural networks against adversarial attacks on medical images. Pattern Recognit..

[43] Cadrin-Chênevert A. (2022). Moving from ImageNet to RadImageNet for improved transfer learning and generalizability. Radiol. Artif. Intell..

[44] Ayeelyan J., Utomo S., Rouniyar A., Hsu H.-C., Hsiung P.-A. (2024). Federated learning design and functional models: A survey. Artif. Intell. Rev..

