# Supplementary material for: Improved Generalizability in Medical Computer Vision: Hyperbolic Deep Learning in Multi-Modality Neuroimaging
Source: J Imaging. 2024 Dec 12;10(12):319. doi: 10.3390/jimaging10120319 (PMC11676359; doi:10.3390/jimaging10120319)
Supplement: Supplementary file 1 [file jimaging-10-00319-s001.zip › Supp2-xml2.pdf]

Supplementary Materials

Table S1. Neuroimaging Class Table Characteristics Tables

Multi-Modality Neuroimaging: Classes	Modality General	Modality Specific	Disease State	Disease Specific
AD Moderate MRI T1	mri	t1	diffuse	ad
AD Severe MRI	mri	t1	diffuse	ad
AD Mild MRI T1	mri	t1	diffuse	ad
Hemorrhagic Stroke Epidural CT Bone	ct	ctbo	lesion	hemm
Hemorrhagic Stroke Intraparenchymal CT Bone	ct	ctbo	lesion	hemm
Hemorrhagic Stroke Intraventricular CT Bone	ct	ctbo	lesion	hemm
Hemorrhagic Stroke Subarachnoid CT Bone	ct	ctbo	lesion	hemm
Hemorrhagic Stroke Subdural CT Bone	ct	ctbo	lesion	hemm
Hemorrhagic Stroke Epidural CT Brain	ct	ctbr	lesion	hemm
Hemorrhagic Stroke Intraparenchymal CT Brain	ct	ctbr	lesion	hemm
Hemorrhagic Stroke Intraventricular CT Brain	ct	ctbr	lesion	hemm
Hemorrhagic Stroke Subarachnoid CT Brain	ct	ctbr	lesion	hemm
Hemorrhagic Stroke Subdural CT Brain	ct	ctbr	lesion	hemm
Ischemic Stroke MRI DWI	mri	dwi	lesion	isch
Ischemic Stroke MRI Flair	mri	flair	lesion	isch
Metastasis MRI Flair	mri	flair	lesion	met
Metastasis MRI T1C	mri	t1c	lesion	met
Metastasis MRI T1	mri	t1	lesion	met
Normal CT Bone	ct	ctbo	normal	normal
Normal CT Brain	ct	ctbr	normal	normal
Normal MRI DWI	mri	dwi	normal	normal
Normal MRI Flair	mri	flair	normal	normal
Normal MRI T1	mri	t1	normal	normal
Normal MRI T1C+	mri	t1c	normal	normal
Normal MRI T2	mri	t2	normal	normal
Schizophrenia MRI DWI	mri	dwi	diffuse	schiz
Schizophrenia MRI T1	mri	t1	diffuse	schiz
Glioma MRI T1C+	mri	t1c	lesion	tumor
Meningioma MRI T1C+	mri	t1c	lesion	tumor
Neurocitoma MRI T1C+	mri	t1c	lesion	tumor
Other Lesions MRI T1C+	mri	t1c	lesion	lesion
Schwannoma MRI T1C+	mri	t1c	lesion	tumor
Glioma MRI T1	mri	t1	lesion	tumor
Meningioma MRI T1	mri	t1	lesion	tumor
Neurocitoma MRI T1	mri	t1	lesion	tumor

Other Lesions MRI T1	mri	t1	lesion	tumor
Schwannoma MRI T1	mri	t1	lesion	tumor
Glioma MRI T2	mri	t2	lesion	tumor
Meningioma MRI T2	mri	t2	lesion	tumor
Neurocitoma MRI T2	mri	t2	lesion	tumor
Other Lesions MRI T2	mri	t2	lesion	lesion
Schwannoma MRI T2	mri	t2	lesion	tumor

† T1 MRI and T1 Contrast Enhanced MRI were designated a 0.5-unit difference. T2 MRI, DWI MRI, and Flair MRIwere designated a 0.5-unit difference. All other difference between a class value was designated as a one-unit difference.

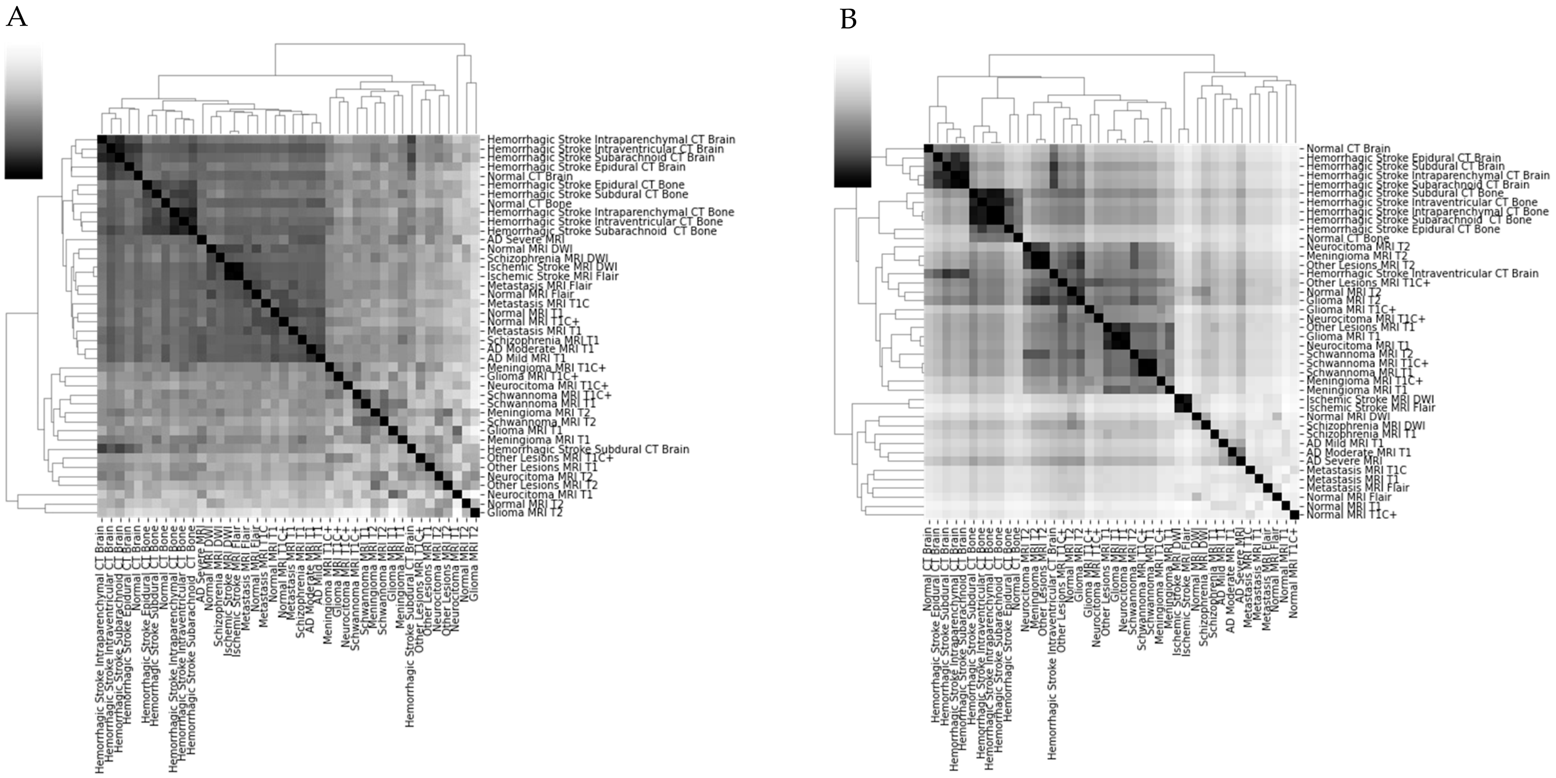

Figure S1. Average Class Embedding Space Distance Heat Map

The figure illustrates the hierarchical clustering dendrogram of the average class embedding space of theEuclidean ResNet 18 (A) and the Euclidean-Lorentz ResNet 18 (B) in the Multi-Modality Neuroimaging(MMN) Dataset. The dendrogram is accompanies by a heatmap where the darker voxels represent a closer distance between the respective classes.

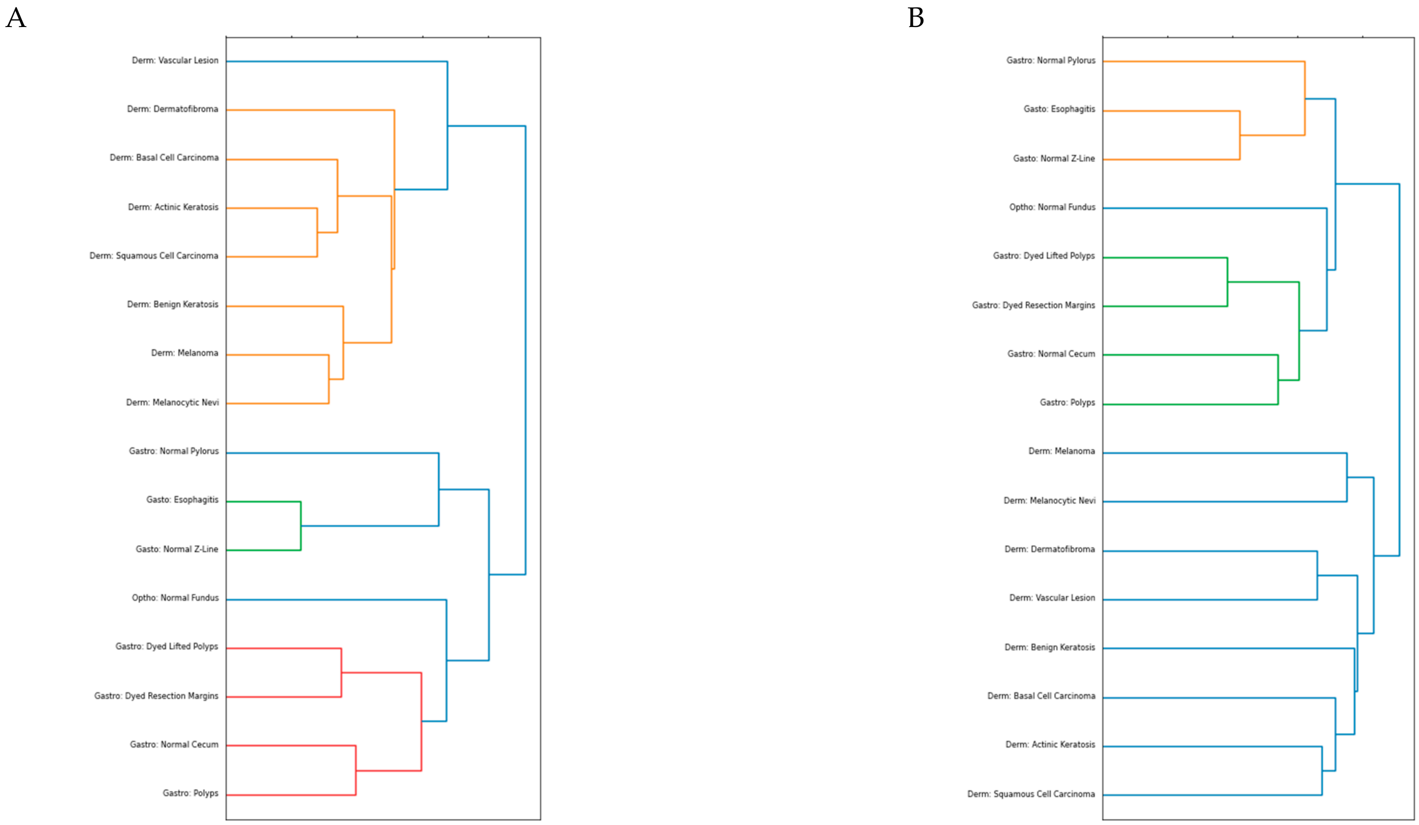

Figure S2. Hierarchical Dendrograms in the Miniature Multi-Disease Dataset

The figure illustrates the hierarchical clustering dendrogram of the average class embedding space of theEuclidean ResNet 18 (A) and the Euclidean-Lorentz ResNet 18 (B) in the Miniature Multi-Disease (MMD) Dataset.

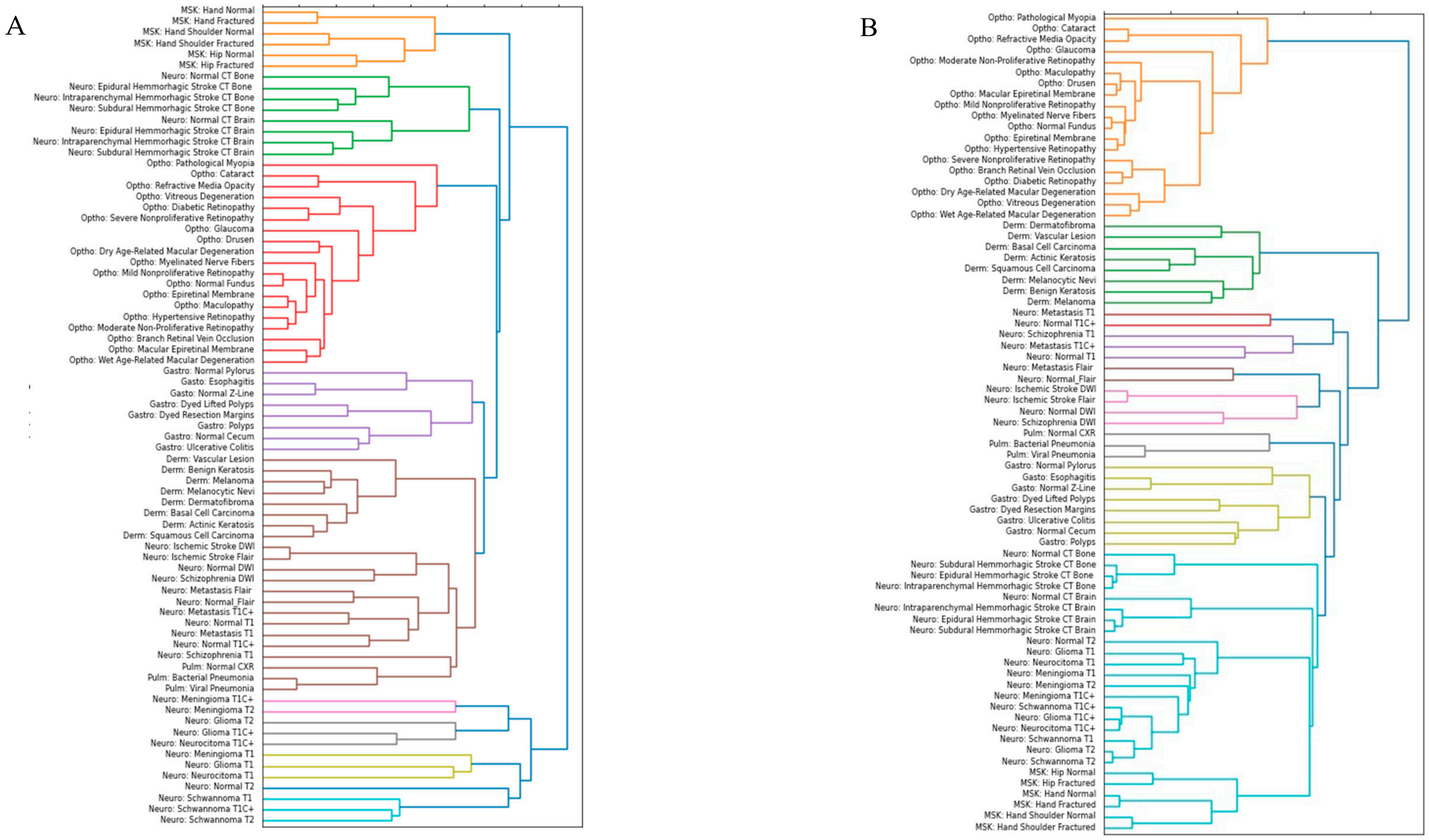

Figure S3. Hierarchical Dendrograms in the Multi-Disease Dataset

The figure illustrates the hierarchical clustering dendrogram of the average class embedding space of theEuclidean ResNet 18 (A) and the Euclidean-Lorentz ResNet 18 (B) in the Multi-Disease (MD) Dataset.
